# Polyfunctional KLRG-1^+^CD57^+^ Senescent CD4^+^ T Cells Infiltrate Tumors and Are Expanded in Peripheral Blood From Breast Cancer Patients

**DOI:** 10.3389/fimmu.2021.713132

**Published:** 2021-07-27

**Authors:** Maria C. Ramello, Nicolás G. Núñez, Jimena Tosello Boari, Sabrina N. Bossio, Fernando P. Canale, Carolina Abrate, Nicolas Ponce, Andrés Del Castillo, Marta Ledesma, Sophie Viel, Wilfrid Richer, Christine Sedlik, Carolina Tiraboschi, Marcos Muñoz, Daniel Compagno, Adriana Gruppi, Eva V. Acosta Rodríguez, Eliane Piaggio, Carolina L. Montes

**Affiliations:** ^1^Departamento de Bioquímica Clínica, Facultad de Ciencias Químicas, Universidad Nacional de Córdoba, Córdoba, Argentina; ^2^Centro de Investigaciones en Bioquímica Clínica e Inmunología (CIBICI-CONICET), Córdoba, Argentina; ^3^PSL Research University, Institut Curie Research Center, Translational Research Department, Paris, France; INSERM U932, Paris, France; ^4^Gynecology Deparment, Hospital Rawson, Córdoba, Argentina; ^5^Laboratory of Molecular and Functional Glyco-Oncology, IQUIBICEN-CONICET-UBA, CABA (Ciudad Autónoma de Buenos Aires), Argentina; ^6^Laboratorio de Medicina experimental y terapéutica, IMIBIO, Universidad Nacional de San Luis, San Luis, Argentina

**Keywords:** senescent T cells, polyfunctional T cells, breast cancer, KLRG-1, CD57, cytotoxic CD4+T cells

## Abstract

Senescent T cells have been described during aging, chronic infections, and cancer; however, a comprehensive study of the phenotype, function, and transcriptional program of this T cell population in breast cancer (BC) patients is missing. Compared to healthy donors (HDs), BC patients exhibit an accumulation of KLRG-1^+^CD57^+^ CD4^+^ and CD8^+^ T cells in peripheral blood. These T cells infiltrate tumors and tumor-draining lymph nodes. KLRG-1^+^CD57^+^ CD4^+^ and CD8^+^ T cells from BC patients and HDs exhibit features of senescence, and despite their inhibitory receptor expression, they produce more effector cytokines and exhibit higher expression of Perforin, Granzyme B, and CD107a than non-senescent subsets. When compared to blood counterparts, tumor-infiltrating senescent CD4^+^ T cells show similar surface phenotype but reduced cytokine production. Transcriptional profiling of senescent CD4^+^ T cells from the peripheral blood of BC patients reveals enrichment in genes associated with NK or CD8^+^-mediated cytotoxicity, TCR-mediated stimulation, and cell exhaustion compared to non-senescent T cells. Comparison of the transcriptional profile of senescent CD4^+^ T cells from peripheral blood of BC patients with those of HDs highlighted marked similarities but also relevant differences. Senescent CD4^+^ T cells from BC patients show enrichment in T-cell signaling, processes involved in DNA replication, p53 pathways, oncogene-induced senescence, among others compared to their counterparts in HDs. High gene expression of CD4, KLRG-1, and B3GAT1 (CD57), which correlates with increased overall survival for BC patients, underscores the usefulness of the evaluation of the frequency of senescent CD4^+^ T cells as a biomarker in the follow-up of patients.

## Introduction

T cell senescence was initially described as a natural process occurring during aging (1). Later, it was described that senescent CD8^+^ T cells were increased in young individuals with chronic infections, autoimmune diseases, and cancer (2–5). Few reports have focused on senescent CD4^+^ T cells, possibly due to the lower susceptibility of this cell population to become senescent in comparison to CD8^+^ T lymphocytes (6). CD4^+^ T cells with senescent features were found in peripheral blood from patients with autoimmune diseases and acute coronary syndrome, as well as during infections with *Trypanosoma cruzi, Leishmania* spp and SARS-COV-2 (7–12).

Using experimental *in vitro* models, we and others demonstrated that tumor cells can trigger in human CD4^+^ and CD8^+^ T lymphocytes the acquisition of suppressive functions and phenotypic alterations that resembled those found in senescent cells (13, 14). CD4^+^ T cells with phenotype compatible with senescence have been also detected in patients with cancer. Thus, aged breast cancer patients receiving chemotherapy showed an accumulation of CD4^+^ T effector memory re-expressing CD45RA (15). In addition, patients with lung cancer exhibited a significant increment of CD4^+^CD28^-^ and CD8^+^ CD28^−^ T cells after chemotherapy compared to healthy donors and untreated patients (16).

The hallmarks of senescent T cells, in particular for CD8^+^ T cells, include: critical shortening of telomeres (17, 18), loss of the expression of the costimulatory molecules CD27 and CD28 (1), CD57 expression (19), increased activity of the lysosomal *β*-galactosidase enzyme (which is called senescence-associated *β*-galactosidase or SA-*β*gal), increased expression of proteins involved in DNA damage responses, such as phosphorylated forms of ATM and *γ*H2AX (20), and expression of the inhibitory receptor (iR) KLRG-1 (21). In addition, senescent T lymphocytes exhibit a cell cycle arrest that is established and maintained by the tumor suppressor pathways p53/p21 and p16/pRB (13, 14, 22, 23).

Senescent CD28^−^CD8^+^ T cells adopt a pro-inflammatory profile characterized by the altered expression of several chemokines and cytokines and their receptors. These cells are able to secrete high levels of pro-inflammatory cytokines, similar to fibroblasts that exhibit the termed senescence-associated secreting phenotype (SASP) (24). The SASP concept, originally described for non-immune cells, remains poorly explored in CD4^+^ and CD8^+^ T cells (23). In this regard, it has been demonstrated that senescent CD8^+^ T cells secrete high levels of TNF, IL-18, and CCL16 among other cytokines and chemokines (25).

To date there are no studies dissecting the functional and transcriptional program of senescent CD4^+^ T cells in cancer patients. In this work, we report that unlike exhausted T cells (26), senescent T cells are increased in peripheral blood of breast cancer (BC) patients and infiltrate tumors and tumor-draining lymph nodes of these patients. In addition, we describe senescent CD4^+^ T cell phenotype and functional and transcriptomic profiles. Finally, we analyze that high gene expression of CD4, KLRG-1, and B3GAT1 (CD57) correlates with increased overall survival for BC patients.

## Materials and Methods

### Human Samples

Whole blood was collected from 24 BC patients and 8 sex and age-matched healthy donors (HDs) (Mean age ± SEM: BC 51.9 ± 2; HD 53.8 ± 2.3, unpaired two-tailed student *t* test: BC *vs* HD age *p* = 0.61). All BC patients recruited did not receive any previous surgery treatment. Blood sampling was done before surgery. Tumors and tumor-draining lymph nodes (LNs) were collected from 30 BC patients from the Institut Curie Hospital (France) and Hospital Rawson (Argentina) (Tumors *n* = 24, LNs *n* = 6). LNs were classified as invaded/metastatic (I-LNs) or non-invaded (NI-LNs) according to the presence of tumor cells determined by histology and confirmed by Epcam/CD45 staining by flow cytometry (see **Supplementary Table S1** for detailed information of the samples of the patients).

Tumors and LNs were mechanically disaggregated and treated with liberase and DNase I (Roche, Buenos Aires, Argentina). Peripheral blood mononuclear cells (PBMCs) were isolated by centrifugation over Ficoll-Hypaque gradients (GE Healthcare, Chicago, IL, USA).

### Flow Cytometry

Single cell suspensions were stained with mAbs against human: CD3, CD8, CD4, CCR7, KRLG1, CD57, CD45RA, CD27, CD28, 2B4, CD160, BTLA, PD-1, TIGIT, and CD107a.

For intracellular staining, cells were fixed/permeabilized with Foxp3 Staining Buffer Kit (eBioscience, San Diego, CA, USA) following manufacturer indications. Fixed human cells were intracellularly stained with anti-human Foxp3, **γ**H2AX, TNF, IFN**γ**, IL-2, Granzyme B, and Perforin.

For intracellular cytokines and Granzyme B detection, cells were stimulated with 50 ng/ml PMA (SIGMA), 1 µg/ml Ionomycin (SIGMA, St. Louis, MO, USA) and Brefeldin A plus Monensin (eBioscience, San Diego, CA, USA) for 4 h at 37°C. For CD107a staining, cells were stimulated as indicated before for 4 h in the presence of anti-CD107a mAb.

For senescence-associated *β*-galactosidase (SA-*β*gal) activity (pH = 6), cells were pre-treated with Bafilomycin A1 (0.1 mM, SIGMA, St. Louis, MO, USA) and then exposed to 5-dodecanoylaminofluorescein di-*β*-D-galactopyranoside (C_12_FDG, Molecular Probes). After 2 h of incubation, cells were washed and then stained with antibodies against surface proteins as previously indicated.

Samples were acquired using BD FACS Canto II, Invitrogen Attune and BD LSR Fortessa flow cytometers, and data were analyzed with FlowJo software. For all analyses in CD4^+^ T cells, Foxp3^+^ regulatory T cells (Tregs) were excluded. According to the gating strategy used for PBMCs (within viable cells), CD8^+^ T cells were defined as CD3^+^CD8^+^, while CD4^+^ T cells were defined as CD3^+^CD8^−^ cells. For tumors (within viable cells), cell subsets were defined as CD8^+^:CD3^+^CD8^+^ and CD4^+^:CD3^+^CD4^+^. Because PMA/Ionomycin stimulation downregulates CD4 expression, for assays which include this stimulation protocol we used the same strategy mentioned for PBMCs for all samples (see **Supplementary Table 2** for detailed information).

### Cell Sorting and *In Vitro* Stimulation

T cells from the peripheral blood of BC patients were pre-purified by using Pan T Selection Kit (Miltenyi, San Diego CA, USA). Then, purified CD3^+^ T cells were stained with CD4-APC, CD8-Alexa700, CCR7-PerCP-Cy5.5, CD45RA-PE-Cy7, CD25-PE, KLRG-1-PerCP-e710, CD57-PE-CF594 and then with DAPI live/dead stain. Stained cells were sorted into CD8^+^ and CD4^+^ effector/memory (CCR7^−^) KLRG-1^−^CD57^−^ (double negative, DN), KLRG-1^+^CD57^−^ (simple positive, SP) and KLRG-1^+^CD57^+^ (double positive, DP) subsets using a FACS Aria IIb Cell Sorter (BD Bioscience, San Jose, CA, USA). Tregs were excluded as CD25hi cells. Sorted cells were stimulated with immobilized anti-CD3 (1 µg/ml, eBioscience, San Diego, CA, USA) and anti-CD28 (0.5 µg/ml, eBioscience, San Diego, CA, USA) for 72 h, and proliferation was determined by Ki-67 expression as described.

### Microarray Analysis

DP and DN CD4^+^ T cells from the blood of three luminal (ER^+^PR^+^HER2^−^) BC patients and three age-matched HDs were sorted based on KLRG-1 and CD57 expression using the following strategy: live CD4^+^CCR7^−^CD25^int/−^ (CD4+, effector/memory and conventional T cells) and lysed with TCL buffer (Qiagen, Les Ulis, France). RNA was isolated with RNA purification kit (Norgen, Ontario, Canada), and its integrity was evaluated with Agilent RNA 6000 pico kit. A 100 ng of RNA was used to synthesize cDNA using reverse-transcription reactions according to the standard Affymetrix protocol. Analyses were performed using Affymetrix Human Gene 2.1 ST arrays. Microarray data have been deposited in the GEO database under accession code: GSE142080.

Gene expression data were normalized using RMA algorithm from oligo package (version 1.42.00) and annotated with manufacturer CDF (version 8.7.0) in R (version 3.4.0). Principal component analysis was performed using R on 29,298 probesets. Probesets with a log2 expression value inferior to 4 in all samples were discarded. After elimination of background, the expression matrix of 10,756 genes was used for downstream analysis. Differentially expressed genes (DEGs) between groups were obtained using the linear model process from the R limma package (version 3.34.8) (27), considering in the model the tumor presence (BC patients and HDs) and the cell status (DP and DN). Only the DEGs with *p*-values ≤0.05 (Benjamini–Hochberg correction at 0.25) have been considered. Heat map of selected genes was produced with Heatmapper (28). Generated DEG lists were analyzed using the HTML5 gene list enrichment analysis tool EnrichR (29, 30); selected pathways and ontologies from specified databases were listed. All gene set enrichment analyses (GSEAs) were run simultaneously using the GSEA software from the Broad Institute (31) with the C2 (curated gene sets) and C7 (immunologic signatures) collections from the Molecular Signatures Database (MSigDB v6.2).

### Expression Analysis From Public Datasets

CD4, KLRG1 and B3GAT1 (as CD57) z-score expressions were extracted from databases available at cBioPortal for Cancer Genomics (32, 33). This portal stores expression data and clinical attributes. The z-score for CD4, KLRG1, and CD57 mRNA expression is determined for each sample by comparing mRNA expression to the distribution in a reference population harboring typical expression for the gene. The query “CD4” or “KLRG1” or “B3GAT1” was performed in all datasets available but only two TCGA datasets for breast invasive carcinoma allow statistical analysis by reaching sufficient patient number for gene level criterions, such as >+1 (high expression) or <−1 (low expression) for each mRNA expression z-scores relative to all samples. The resulting number of each patient population in accordance with the level of mRNA expression is indicated in the corresponding figures. The mRNA expression from selected data was plotted in relation to the clinical overall survival in each sample, and overall survival was calculated when the survival of the selected population reaches 50%. Log rank Test p and q as corrected significance were used as statistical analyses.

### Statistical and Bioinformatics Analysis

GraphPad Prism software was used for statistical analysis. For comparisons of HD and BC patients, unpaired two-tailed Student’s *t*-tests or one-way ANOVA with Sidak’s post-test was used. For T cell subset comparisons (within HD or BC group) paired two-tailed Student’s *t*-tests or repeated measures one-way ANOVA with Tukey’s post-test was used. Graphs always represent mean ± SEM. *p-*values <0.05 were considered statistically significant.

## Results

### KLRG-1^+^CD57^+^ CD4^+^ and CD8^+^ T Cells Are Increased in Peripheral Blood and Infiltrated Tumors and Lymph Nodes From Breast Cancer Patients

**Figure 1:**
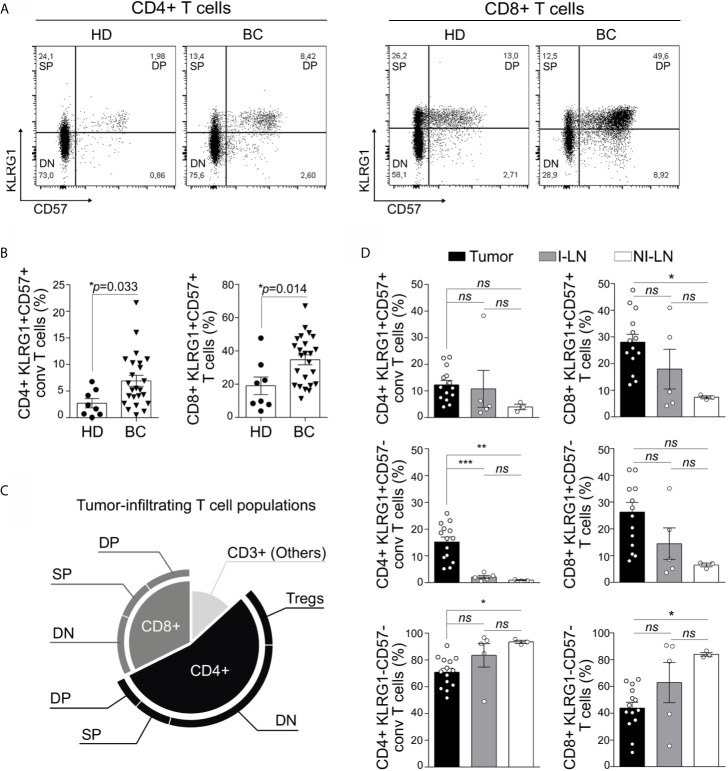
KLRG-1^+^CD57^+^ CD4^+^ and CD8^+^ T cells are present in peripheral blood, tumor, and invaded and non-invaded lymph nodes from untreated breast cancer patients. **(A)** Representative dot plots from one healthy donor (HD) and one breast cancer patient (BC), show the frequency of peripheral blood (PB) CD4^+^ and CD8^+^ T cells based on the surface expression of KLRG-1 and CD57 molecules. **(B)** Bar graphs show frequency of KLRG-1^+^CD57^+^ CD4^+^ T cells and KLRG-1^+^CD57^+^ CD8^+^ T cells from PB of HDs (dots) and BC patients (triangles). Each dot/triangle represents a subject analyzed. Unpaired Student’s *t*-test was used to compare double positive CD4^+^ or CD8^+^ T cells in HD *vs.* BC patients (*p* is indicated in each graph). **(C)** Proportion of CD4^+^ and CD8^+^ subsets within tumor-infiltrating CD3^+^ population. **(D)** Bar graphs show mean frequency (± SEM) of KLRG-1^+^CD57^+^ (DP), KLRG-1^+^CD57^−^ (SP), KLRG-1^−^CD57^−^ (DN) within conventional CD4^+^ (left panel) or CD8^+^ (right panel) T cells in tumor (black), invaded lymph nodes I-LN (gray) and non-invaded LN, NI-LN (white). One-way ANOVA with Sidak’s post-test was applied. *p < 0.05, **p < 0.01, ***p < 0.001, ns, not significant.

KLRG-1 and CD57, two cell surface NK receptors, have been used separately as markers of human senescent T cells (19, 21). We used these markers to evaluate the presence of senescent T cells in peripheral blood, tumors, invaded (I) and non-invaded (NI) tumor-draining lymph nodes (TDLNs) from newly diagnosed patients with BC having undergone standard of care surgical resection. Thus, we identified three major subpopulations within circulating conventional (Foxp3^−^) CD4^+^ and CD8^+^ T cells: KLRG-1^−^CD57^−^ T cells (double negative, DN), KLRG-1^+^CD57^−^ T cells (single positive, SP), and KLRG-1^+^CD57^+^ T cells (double positive, DP) (**Figure 1A**). When compared to age-matched HDs, BC patients show increased percentages of KLRG-1^+^CD57^+^ (DP) cells within CD4^+^ and CD8^+^ populations in peripheral blood (**Figure 1B**).

We next evaluated the subset-based composition of tumor-infiltrating T cells. We found greater proportion of CD4^+^ than CD8^+^ T lymphocytes infiltrating untreated BC tumors. Although DN subsets were predominant within CD4^+^ as well as CD8^+^ cells, a relevant fraction (comparable in frequency to Tregs) of these tumor-infiltrating CD4^+^ and CD8^+^ T cells exhibited the DP or SP phenotype (**Figure 1C**). We also detected DP and SP CD4^+^ and CD8^+^ T cell subsets infiltrating invaded (I) and non-invaded (NI) TDLNs (**Figure 1D**). Comparison of the different subsets among tumors, I and NI TDLNs showed no significant differences in the frequency of DP CD4^+^ T cells. Differently, the frequency of SP CD4^+^ T cells was higher in tumor than in I or NI TDLNs while DN CD4^+^ T cells showed significant differences only between tumor and NI TDLNs. Within the CD8^+^ compartment, we detected differences in frequencies of DP and DN CD8^+^ T cells among tumor and NI TDLNs, while no differences were found in frequencies of SP CD8^+^ T cells in any of the tissues evaluated (**Figure 1D**). Interestingly we found that CD4^+^ as well CD8^+^ DN T cells were decreased in tumors with respect to NI TDLNs (**Figure 1D**).

Herein we report that CD4^+^ and CD8^+^ T cells co-expressing KLRG-1 and CD57 were present in peripheral blood, tumor, and invaded and non-invaded lymph nodes from patients with untreated BC.

### The Co-Expression of KLRG-1 and CD57 Defines a T Cell Population With Features of Senescence

**Figure 2:**
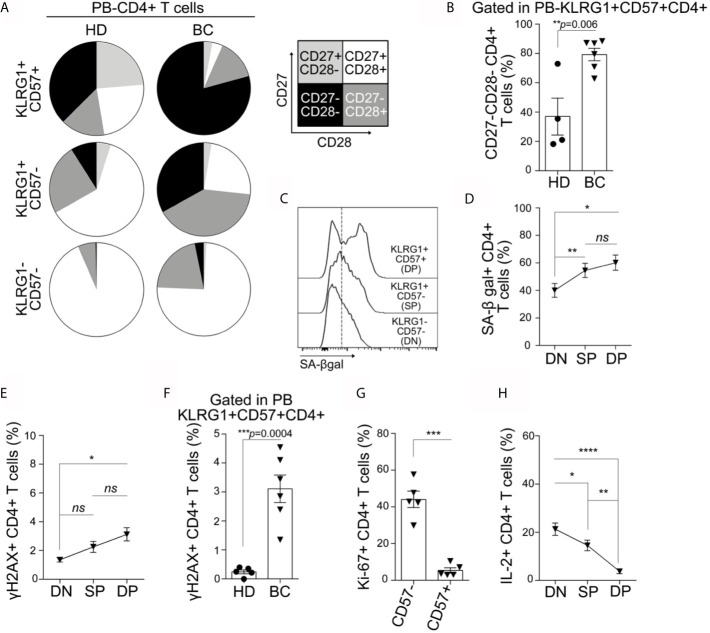
KLRG-1^+^CD57^+^ CD4^+^ cells from peripheral blood exhibit features of senescence. **(A)** Pie charts exhibit proportion of CD27^+^CD28^+^ (white), CD27^+^CD28^-^ (light gray), CD27^−^CD28^+^ (dark gray) and CD27^−^CD28^−^ (black) within CD4^+^ T cell subpopulations defined by the expression of KLRG-1 and CD57, for all HDs and BC patients analyzed. **(B)** Bar graph shows frequency of CD27^−^CD28^−^ cells within KLRG-1^+^CD57^+^ CD4^+^ T cells from HDs (dots) and BC patients (triangles). **(C)** Representative histograms show the expression/activity of SA-*β*gal in KLRG-1^−^CD57^−^ (DN), KLRG-1^+^CD57^−^ (SP) and KLRG-1^+^CD57^+^ (DP) CD4^+^ T cells from BC patients. **(D)** Mean frequency (± SEM) of SA-βgal^+^ CD4^+^ T cells within DN, SP and DP subsets. **(E)** Mean frequency (± SEM) of **γ**H2AX^+^ cells in DN, SP and DP CD4^+^ T cell subsets. **(F)** Bar graph shows frequency of **γ**H2AX^+^ cells within KLRG-1^+^CD57^+^ CD4^+^ T cells from HDs (dots) and BC patients (triangles). **(G)** Bar graph shows frequency of proliferating (Ki-67^+^) CD4^+^ T cells after stimulation and within CD57^−^ and CD57^+^ populations from BC patients. **(H)** Mean frequency (± SEM) of IL-2^+^ cells after PMA/Ionomycin stimulation in DN, SP, and DP CD4^+^ T cells. In all cases, each dot/triangle/square represents a subject analyzed. For two group comparisons (HD *vs* BC) unpaired Student’s *t*-tests were used in all cases (*p* is indicated in each graph). For more three groups comparisons (DN, SP, and DP) matched one-way ANOVA and Tukey multiple comparison tests were used (*ns*, not significant; *p < 0.05; **p < 0.01; ***p < 0.005; ****p < 0.001).

We next analyzed within the CD4^+^ T cell population other markers commonly associated with T cell senescence such as loss of costimulatory molecules (CD28 and CD27), high activity of SA-*β*gal, DNA damage, and cell cycle arrest (6). We found that DP CD4^+^ T cell subset contained the highest proportion of CD27^−^CD28^−^ cells compared to the SP and DN counterparts in both BC patients and HDs (**Figure 2A**). In addition, SP T cells from BC patients displayed an accumulation of CD27^−^CD28^−^ cells compared to the DN subset. Of note, DP CD4^+^T cells from BC patients exhibited the majority of the cells with the CD27^−^CD28^−^ phenotype, which is significantly higher than their counterparts from HDs (**Figure 2B**). Within CD4^+^ T cells from BC patients, SA-*β*gal activity was similar in the DP and SP cells but significantly higher in these subsets compared to the DN cells (**Figures 2C, D**). No significant differences were observed in SA-*β*gal activity among the T cell subsets from HDs and BC patients (*data not shown*). Regarding the expression of *γ*H2AX, a DNA damage response-related phosphorylated histone, we determined that DP CD4^+^ cells from BC patients exhibited the higher frequency of cells expressing this protein in comparison to the DN T subset from BC patients (**Figure 2E**) as well as the DP subset from HDs (**Figure 2F**). We next evaluated the proliferative response of CD4^+^ T cells from BC patients. Because KLRG-1 expression was completely downregulated after stimulation of PBMCs with anti-CD3/CD28 beads, proliferation was first analyzed in CD57^−^ *versus* CD57^+^ cells. We found that CD57^+^CD4^+^ T cells exhibited reduced proliferation capacity compared to CD57^−^ counterparts, as evidenced by the lower frequency of Ki-67^+^ cells (**Figure 2G**). To confirm this finding in all the subsets considering KLRG-1 expression, DN, SP, and DP T cell subsets were sorted from BC patients and stimulated for 72 h with anti-CD3/CD28 beads. The analysis of Ki-67 expression confirmed that DP and SP CD4^+^ cells failed to proliferate under these conditions (**Supplementary Figure S1A**). Accordingly, the frequency of IL-2-expressing cells was significantly reduced in CD4^+^ DP and SP subsets compared to DN subsets (**Figure 2H**).

We also evaluated the CD8^+^ T cells from peripheral blood from BC patients and HDs. We observed that the phenotype and functional behavior of DP CD8^+^ T cells were similar to that of DP CD4+T. In addition, the differences between DP CD4^+^ from BC patients and HDs concerning the increased frequency of CD27^−^CD28^−^ and *γ*H2AX^+^ were also detected in the DP CD8^+^ T cell compartment (**Supplementary Figures S2A–G**).

Our results demonstrated that co-expression of KLRG-1 and CD57 defines a population with attributes of senescent T cells. By using these markers, we were able to define not only senescent CD8^+^ T cells, but also not previously described senescent conventional CD4^+^ T cells in peripheral blood and tissues of untreated patients with BC.

### Senescent T Cells From Peripheral Blood Exhibit an Effector Memory/EMRA Phenotype

We next evaluated the differentiation profile of CD4^+^ and CD8^+^ T cells from peripheral blood of BC patients and HDs using the combination of CCR7 and CD45RA markers to define: effector memory [CCR7^−^CD45RA^−^, (EM)], effector memory expressing CD45RA [CCR7^−^CD45RA^+^, (EMRA)], central memory [CCR7^+^ CD45RA^−^, (CM)], and naïve [CCR7^+^CD45RA^+^, (N)]. We observed that, except for naïve CD4^+^ T cells that were reduced in BC patients, there were no differences between HDs and BC patients in the frequency of T cell subpopulations within CD4^+^ and CD8^+^ T cells (**Supplementary Figure S3**). These data show that the increased percentage of DP senescent T cells in BC patients with respect to HDs is not due to an increase in EM or EMRA subsets.

**Figure 3:**
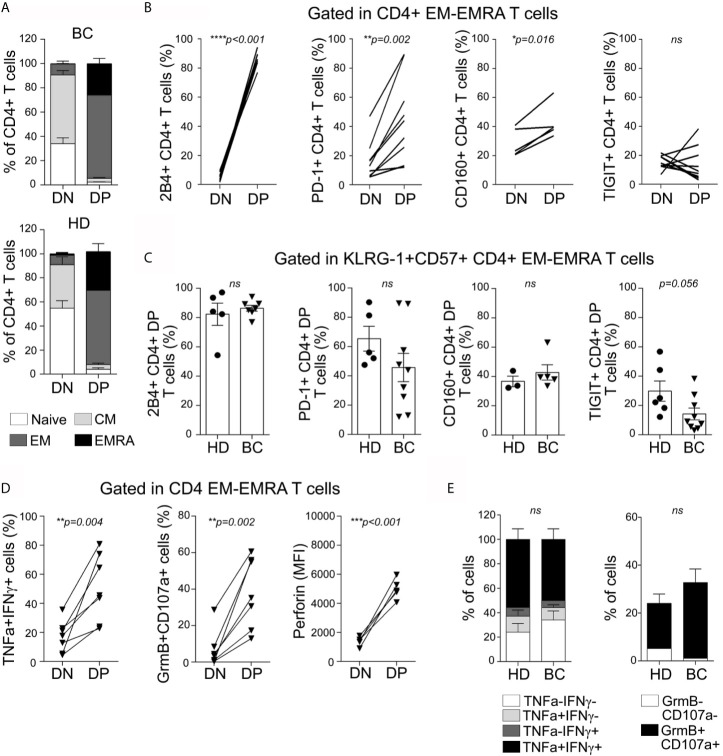
Senescent CD4^+^ T cells from peripheral blood exhibit an effector-memory/EMRA phenotype, express high levels of inhibitory receptors, and are highly polyfunctional. **(A)** Bar graphs show proportion of naive (white), CM (light gray), EM (dark gray), and EMRA (black) cells within DN and DP CD4^+^ T cell subpopulations, for all BC patients (top) and HDs (bottom) analyzed. **(B)** Line graphs show frequency of iR-expressing CD4^+^ T cells within EM/EMRA populations and within DN and DP subsets as indicated in graphs. Each line represents a subject analyzed. **(C)** Bar graphs show iR-expressing CD4^+^ T cells within KLRG-1^+^CD57^+^ EM/EMRA population from BC patients and HDs. Unpaired *t*-tests were used to compare iR expression between DN and DP subsets in each sample (HD or BC). **(D)** Line graphs show frequency of BC patients’ CD4^+^ T cells co-expressing TNF and IFN**γ** or Granzyme B and CD107a (after PMA/Ionomycin stimulation) or expressing Perforin (*ex vivo*), within EM/EMRA populations and within DN and DP subsets as indicated in graphs. Each line represents a subject analyzed. Paired *t*-tests were used to compare functionality between DN and DP subsets (*p* are indicated in each graph). **(E)** Bar graphs show proportion of KLRG-1^+^CD57^+^CD4^+^ T cells expressing the indicated cytokines or cytotoxic-associated molecules in HDs and BC patients. Unpaired *t*-test was applied to compare HD *vs* BC (ns, not significant).

To further characterize the circulating DN, and DP CD4^+^ T subsets, we examined their activation/differentiation status according to the expression of markers mentioned above. We concluded that DP CD4^+^ T cells from both groups (BC patients and HDs) were predominantly EM or EMRA cells, while naïve and CM phenotypes were more represented in DN cells (**Figure 3A**). Similarly, most of DP CD8^+^ T cells from BC patients and HDs showed an EM or EMRA phenotype (**Supplementary Figure S4A**).

### Senescent CD4^+^ T Cells From Peripheral Blood Express Multiple Inhibitory Receptors

Beyond KLRG-1 or Tim-3, it has not been reported whether senescent T cells from cancer patients co-express other iRs. To evaluate the expression of different iRs on the DP and DN CD4^+^ T cells from peripheral blood while avoiding misleading comparisons as a consequence of the marked differentiation heterogeneity (**Figure 3A**), we restricted the analysis to cells that showed an EM/EMRA phenotype. More than 80% of DP CD4^+^ T cells (82 ± 17%) from BC patients expressed the iR 2B4; the frequency was significantly higher than that observed within the DN T cell subset (**Figure 3B**). 2B4-expressing cells within DN CD4^+^ T cells from BC patients were nearly undetectable, suggesting that this iR could also contribute to the identification of senescent CD4^+^ T cells. In addition, we observed that DP CD4^+^ T cells exhibited higher expression of PD-1, and CD160 but not TIGIT in comparison to DN CD4^+^ T cells (**Figure 3B**). There were no differences between DP CD4+T from BC patients and HDs regarding the expression of 2B4, PD-1, CD160, and TIGIT (**Figure 3C**).

Similar to CD4^+^ T cells, DP CD8^+^ T cells exhibit higher expression of 2B4, PD-1, TIGIT, and CD160 than DN counterparts, and except for TIGIT expression, no differences were detected between DP CD8+T from BC patients and HDs (**Supplementary Figures S4B, C** **)**.

Overall, our data demonstrated that DP CD4^+^ and CD8^+^ T cells from both BC patients and HD expressed iRs such as 2B4, PD-1, and CD160. These results suggest that iR expression by DP T cells might not only be influenced by chronic stimulation as it occurs in the context of a tumor but might also be associated with a complex senescence program.

### Senescent T Cells From Peripheral Blood Show a Polyfunctional Effector Phenotype

We next analyzed production of cytokines and expression of cytotoxicity-associated molecules in senescent CD4^+^ T cells with an EM/EMRA phenotype from peripheral blood of BC patients and HDs. After PMA/Ionomycin stimulation, DP CD4^+^ T cells exhibited higher frequency of TNF^+^ IFN*γ* ^+^ cells and GranzymeB^+^CD107a^+^ cells than the DN counterparts (**Figure 3D**). Moreover, DP T cells showed the highest intracellular expression of Perforin *ex vivo*. No significant differences were observed between DP CD4^+^ from BC patients and HDs regarding cytokine production or Granzyme/CD107a expression (**Figure 3E**). Similarly, DP CD8^+^ T cells exhibited higher frequency of TNF^+^IFN*γ* ^+^ and Granzyme B^+^CD107a^+^ cells as well as higher Perforin expression than DN CD8^+^ T cells, and no significant differences were detected between subsets from BC patients and HDs (**Supplementary Figure S4D, E** **)**.

These data demonstrated that DP CD4^+^ and CD8^+^ T cells from peripheral blood of untreated BC patients and HDs exhibit an effector phenotype. Remarkably, we observed that, resembling CD8^+^ T cells, DP CD4^+^ T cells showed a highly cytotoxic potential that may be relevant in the response against tumors.

### Polyfunctional Senescent T Cells Infiltrate Primary Breast Tumors

**Figure 4:**
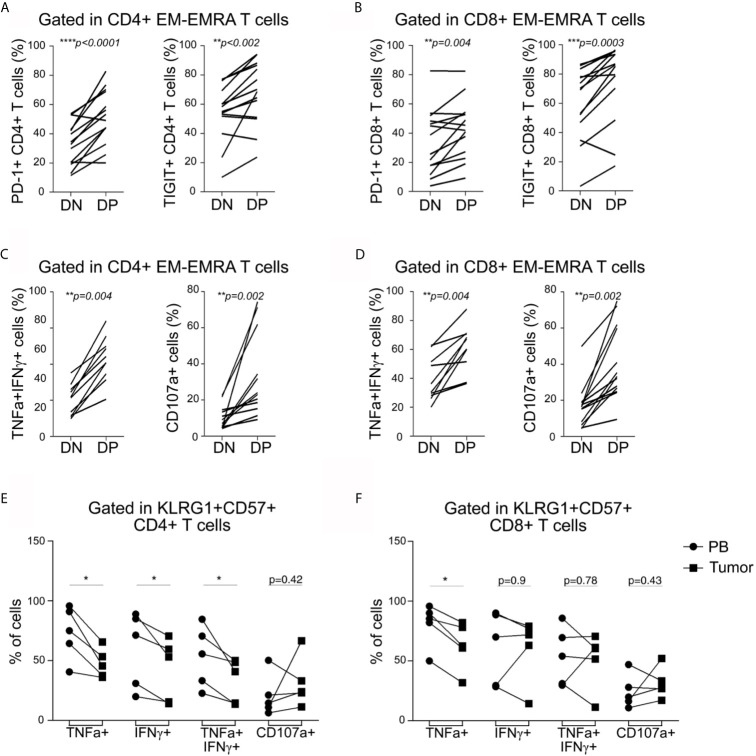
Tumor-infiltrating CD4^+^ and CD8^+^ T cells express inhibitory receptors and are highly polyfunctional cells. **(A, B)** Frequency of tumor-infiltrating CD4^+^ **(A)** and CD8^+^ **(B)** T cells expressing PD-1 or TIGIT within EM/EMRA populations and within DN and DP subsets as indicated in graphs. Each line represents a sample analyzed. **(C, D)** Frequency of tumor-infiltrating CD4^+^ **(C)** and CD8^+^ **(D)** T cells co-expressing TNF and IFN**γ** or expressing CD107a within EM/EMRA populations and within DN and DP subsets as indicated in graphs. Each line represents a sample analyzed. Paired *t*-tests were used to compare DN and DP subsets (*p* are indicated in each graph). **(E, F)** Frequency of cytokine- or CD107a-expressing senescent CD4^+^ **(E)** and CD8^+^ **(F)** T cells within EM/EMRA populations in peripheral blood (PB, circles) *vs* tumor (squares). Each line represents a paired PB–tumor from the same patient. Paired *t*-tests were used to compare both tissues (PB *vs.* T: *p < 0.05).

Similar to peripheral blood counterparts, senescent CD4^+^ and CD8^+^ T cells from tumors were mostly EM/EMRA cells (*data not shown*). Furthermore, the DP EM/EMRA CD4^+^ and CD8^+^ T subsets in the tumor exhibited a significantly higher frequency of PD-1 and TIGIT-expressing T cells compared to DN T cells (**Figures 4A, B** **)**. Senescent CD4^+^ and CD8^+^ T cells in tumors also retained a high capacity to produce effector cytokines and to degranulate compared to other non-senescent T cell subsets (**Figures 4C, D** **)**. Similar functional features were observed for senescent CD4^+^ T cells that infiltrate I-TDLNs (**Supplementary Figure S5A** and data not shown). By comparing the effector function of senescent T cells from tumor and blood, we determined that regardless of their polyfunctional phenotype, tumor-infiltrating senescent CD4^+^ T cells exhibited a reduced effector function as highlighted by the lower frequency of cytokine-producing cells (**Figure 4E**). In contrast, no significant differences were observed in the frequencies of cytokine-producing cells (except for TNF^+^ cells) senescent CD8^+^ T cells in blood and tumor (**Figure 4F**).

We concluded that, despite showing cell cycle arrest and iR expression, senescent CD4^+^ and CD8^+^ T cells (in all tissues evaluated) have a polyfunctional phenotype characterized by coproduction of effector cytokines and cytotoxic molecules. However, unlike senescent CD8^+^ T cells, senescent CD4^+^ T cells accumulated within tumors are less functional than circulating counterparts, suggesting that senescent CD4^+^ T cells are more susceptible to functional loss than CD8^+^ T cells.

### Transcriptional Profiling of CD4^+^ Senescent T Cells From Peripheral Blood

**Figure 5:**
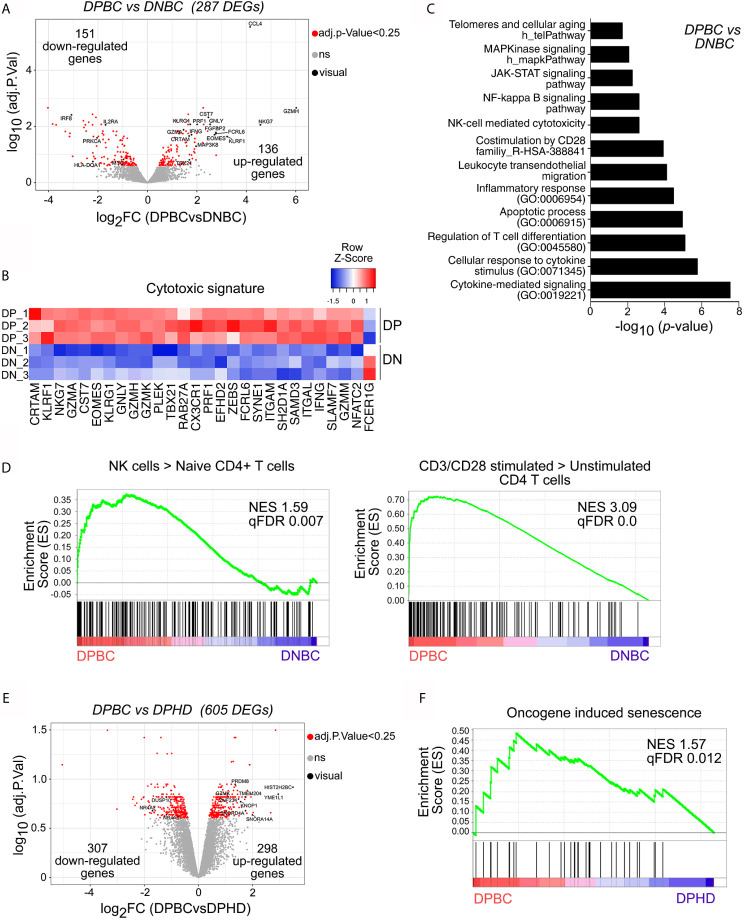
CD4^+^ senescent T cells from breast cancer patients exhibit a distinctive transcriptional profile. Affymetrix microarray analysis performed in sorted KLRG-1^+^CD57^+^ and KLRG-1^−^CD57^−^ CD4^+^ T cells from BC patients (DPBC and DNBC, respectively) and KLRG-1^+^CD57^+^ CD4^+^ T cells from HDs (DPHD). **(A)** Volcano plot shows differentially expressed genes (DEGs) between DPBC and DNBC samples (*p-*value <0.05 corrected with Benjamini–Hochberg, indicated as red dots). Genes of interest are shown in black with their gene symbols. **(B)** Heat map shows the normalized gene expression of cytotoxic signature. **(C)** Graph shows the p-value of selected pathways significantly enriched in DEGs of panel A (DPBC *vs* DNBC) using EnrichR. **(D)** GSEA enrichment plots shows selected gene sets (GSE: 22886 and GSE: 45739) enriched in DPBC *vs* DNBC. Normalized enrichment score (NES) and *p-*value (FCRq) are indicated. **(E)** Volcano plot shows DEGs between DPBC and DPHD samples (*p-*value < 0.05 corrected with Benjamini–Hochberg, indicated as red dots). Selected genes are highlighted in black. **(F)** GSEA plot shows the enrichment of the gene set Reactome oncogene-induced senescence in the transcriptome of DPBC *vs* DPHD. Significant *p-*value (FCRq) is indicated.

To study the transcriptional profile of BC-associated senescent CD4^+^ T cells, we performed a microarray assay of peripheral blood cell subsets sorted as KLRG-1^+^CD57^+^ EM/EMRA CD4^+^ cells (DPBC) and KLRG-1^−^CD57^−^ EM/EMRA CD4^+^ cells (DNBCs) from treatment-free BC patients and KLRG-1^+^CD57^+^ EM/EMRA CD4^+^ cells from HDs (DPHD). Principal component analysis (PCA) showed that the three CD4^+^ T cell subsets analyzed clustered separately, reflecting their differential transcriptional profiles (**Supplementary Figure 6A**). To define the molecular profile of BC senescent CD4^+^ T cells, we searched for the differentially expressed genes (DEGs) between DPBC and DNBC samples and found 136 and 151 significantly up- and downregulated DEGs, respectively, in the DPBC subset (**Figure 5A**). As annotated in the volcano plot, genes encoding products related with cytotoxicity such as GZMH, GZMA, PRF1 and IFNG, EOMES, TBX21, and CRTAM were upregulated in DPBC cells, highlighting their cytotoxic phenotype. Furthermore, the heat map illustrated selected genes involved in NK- or CD8^+^-mediated cytotoxic processes that were upregulated in DPBC compared to DNBC samples (**Figure 5B**). Biological pathway analysis using EnrichR indicated that, in comparison to DNBC, DPBC were enriched in cytokine-mediated signaling, NF-kappa B, JAK-STAT, and MAPK pathways, as well as in pathways and processes commonly associated with senescence such as CD28-mediated costimulation, apoptosis, inflammatory responses, and telomere-associated aging (**Figure 5C**). Moreover, genes involved in chemokine/cytokine networks as well as T-cell signal transduction were differentially represented between DPBC and DNBC cells (**Supplementary Figure 6B**). Gene set enrichment analysis (GSEA) revealed that the transcriptional signature of DPBC cells was enriched in genes associated with NK-signature and T cell activation (**Figure 5D**). Furthermore, we confirmed a significant enrichment in gene signatures associated with T-cell exhaustion in DPBC cells (**Supplementary Figure S6C**), suggesting that some of the processes regulated in senescent T cells from BC patients were driven by TCR-mediated signals.

We next compared the transcriptomes of circulating DP CD4^+^ T cells from BC and HD that showed marked similarities at phenotypic level (**Figures 2**, **3**). Of note, transcriptome analysis highlighted that 298 genes were significantly upregulated, and 307 genes were significantly downregulated in DPBC compared to their counterparts in HDs (**Figure 5E**). In addition, we observed that DP BC showed enrichment in pathways associated with T-cell signaling and processes involved in catabolism, RNA binding and processing, DNA replication and p53 pathways, all transcriptional profiles associated with senescence (**Supplementary Figure S6D**). These results, together with the GSEA analysis showing that DPBC were enriched in genes associated with PD-1^high^ subsets (**Supplementary Figure S6E**) suggested that senescent CD4^+^ T cells in BC patients exhibited a more activated profile compared to their counterparts in HDs. Interestingly, GSEA analysis revealed that the gene expression profile of DPBC cells was enriched in genes associated with oncogene-induced senescence, when compared with the DPHD transcriptome (**Figure 5F**). Differently, the aging-associated gene set was not significantly enriched in any of the two senescent populations (**Supplementary Figure S6F**). These results suggested that DP from BC and HD had both undergone senescence processes; though, the mechanism of induction and/or maintenance could be different in both conditions. We observed that tumors did not induce significant changes on the phenotype of senescent T cells; however, some molecular programs related to DNA reparation, T-cell costimulation markers or oncogene-induced senescence were modified in DP CD4^+^ T cells from BC patients compared to HDs, suggesting that signals derived from tumors may influence certain biological functions of this T cell subset.

Overall, transcriptional analysis of senescent CD4^+^ T cells from untreated BC patients revealed an enrichment in genes associated with cytotoxicity, T cell activation, and T cell exhaustion compared to non-senescent T cells. Comparison of the transcriptional profile of senescent CD4^+^ T cells from BC patients with that of HDs highlighted marked similarities but also relevant differences indicating that tumors may trigger processes that influenced senescence in T cells.

### High Expression of CD4^+^KLRG-1^+^CD57^+^ Correlated With Increased Overall Survival for Breast Cancer Patients

**Figure 6:**
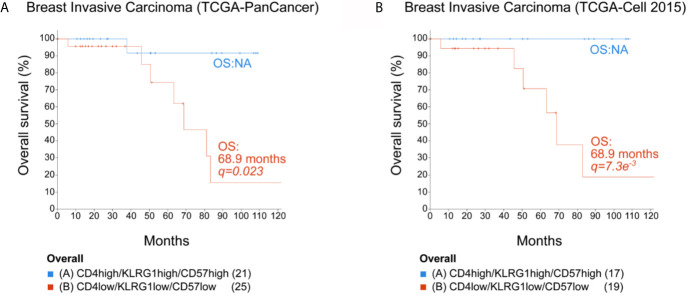
Overall survival for breast cancer patients. Expression data from two TCGA breast invasive carcinoma cohorts were analyzed using cBio Cancer Genomics Portal (http://cbioportal.org). The overall survival (OS) of patients was calculated in accordance with the level of mRNA expression (low or high) of CD4, KLRG1, and B3GAT1 (CD57) (mRNA expression z-scores relative to all samples (logRNAseqs V2 RSEM). **(A)** TCGA-PanCancer Cohort. **(B)** TCGA-Cell 2015 cohort. NA, not analyzed when less than 50% of the patients died. Statistical analysis by log rank Test p and q. The number of patients in each selected population is indicated in parenthesis in each KM plot.

To explore the possible contribution of the KLRG1^+^CD57^+^ CD4^+^ T cell subset in the clinical prognosis of breast cancer, we collected publicly available TCGA datasets of patients with breast carcinoma to link gene expression data with overall survival (OS). For this study, we analyzed the mRNA expression for CD4, KLRG-1, and B3GAT1 (CD57) to define a senescent CD4+T signature. We used the increased or decreased expression (represented as high or low, respectively) of these three genes as the value of the mRNA expression for each of these cell markers compared to the mean of the corresponding expression for each patient included in the analyzed cohort. The lower expression of the signature genes was found positively correlated with a lower survival rate when we analyzed the OS of patients from two invasive breast carcinoma cohorts from the TCGA consortium (TCGA-PanCancer, and TCGA-Cell 2015) (**Figures 6A, B** **)**. These data showed that the simultaneous high expression of CD4, KLRG1, and CD57 within breast tumors correlated with better prognosis.

## Discussion

Accumulation of tumor-induced senescent CD8^+^ T cells has been described in peripheral blood of patients with different types of cancer (5, 34, 35). Here, we show that patients with untreated breast tumors exhibit an accumulation of not only CD8^+^ but also CD4^+^ senescent T cells in peripheral blood. Furthermore, these senescent T cells infiltrate tumors as well as invaded/metastatic and non-invaded tumor-draining lymph nodes. To our knowledge, this is the first demonstration that senescent CD4^+^ T cells are accumulated in young female breast cancer patients who did not receive any treatment.

For many years, loss of CD28 and CD27 expression has been the hallmark of T cell senescence (6). More recently, senescent T cells have been identified by the expression of KLRG-1 or CD57 in the context of aging or viral infection (21, 36). We show that the co-expression of KLRG-1 and CD57 also constitutes a useful tool for the identification of CD4^+^ as well as CD8^+^ senescent T cells in peripheral blood from BC patients. The KLRG-1^+^CD57^+^ (DP) T cell subpopulation exhibits the main features of senescent T cells, such as loss of CD28 and CD27 expression and *γ*H2AX, higher SA-*β*gal activity as well as reduced ability to produce IL-2 and to proliferate in response to polyclonal stimulation. Interestingly, KLRG-1^+^ (SP) CD4^+^ T cells exhibit intermediate features between senescent (DP) cells and non-senescent (DN) cells in terms of proportion of CD27^−^CD28^−^ cells and frequency of SA-*β*gal and IL-2^+^ expressing cells. Consequently, we propose that the single expression of KLRG-1 in the CD4^+^ T cell pool may distinguish a pre-senescent population that displays some of the characteristics associated with senescence. Furthermore, our findings underscore the limitation of the use of KLRG-1 alone as marker of senescent CD4^+^ T cells.

Despite the extensive use of KLRG-1 as a marker of differentiation (37), functional analysis highlights that KLRG-1 may play an inhibitory role in T cells. Indeed, the cross-linking of TCR and KLRG-1 induced in murine T cells decreases IL-2 production (38). In addition, Henson et al. (21) demonstrated that defective Akt (Ser^473^) phosphorylation and proliferation of highly differentiated CD28^−^CD27^−^ CD8^+^ T cells are actively regulated by KLRG-1 signaling and can be reversed by blocking the interaction of this molecule with its ligand, E-cadherin. Thus, the inhibitory role of KLRG-1 may explain the defect in IL-2 production and proliferative activity that we observed in KLRG-1^+^CD57^+^ CD4^+^ and CD8^+^ T cells. Remarkably, KLRG-1^+^CD57^+^ CD4^+^ and CD8^+^ T cells from BC patients exhibited high expression of iRs previously associated with EM/EMRA phenotypes such as 2B4, CD160 and PD-1. We speculate that the expression of these iRs on KLRG-1^+^CD57^+^ T cells may also contribute to the arrest in the cell cycle and the reduced ability to produce IL-2.

Several studies have demonstrated that senescent and exhausted T cells comprise different subpopulations (39). Initially, senescent T cells were described as T cells that lost the expression of the costimulatory molecules CD28 and CD27, and expressed markers associated with replicative senescence such as KLRG-1 and CD57; while exhausted T cells were identified by a sustained co-expression of multiple inhibitory receptors and, as we recently reported, CD39 (26). Furthermore, senescence has been associated with the ability to produce pro-inflammatory cytokines (SASP profile) together with an inability to proliferate (24). Differently, exhausted T cells are incapable of proliferating and exhibit impaired cytokine production (40). In this work, we demonstrate that senescent CD4^+^ and CD8^+^ T cells from both BC patients and HDs share some features with exhausted T cells including cell cycle arrest and iR expression. Furthermore, CD4^+^ senescent T cells exhibit a transcriptional profile enriched in exhaustion-associated genes. Notably, we observed that, different to exhausted T cells which are absent in peripheral blood from patients with breast cancer (26), senescent CD4^+^ and CD8^+^ T cells are expanded in circulation. Additionally, we found that senescent CD4^+^ T cells are enriched in genes associated with NK-mediated cytotoxicity when compared with non-senescent CD4^+^ T cells, suggesting an innate-like ability to kill tumors.

Senescent CD4^+^ T cells from BC patients and HDs exhibit high similarity in phenotype and *ex vivo* functionality, suggesting that tumors induce accumulation/expansion of this population without distinguishable changes in their function. However, transcriptomic analysis of DPBC *vs* DPHD CD4^+^ T cells indicated that the induction of a senescence program in CD4^+^ T cells might be different in cancer patients compared to HDs. In this regard, DPBC cells were enriched in oncogene-induced senescence pathways, but no differences were found in cell aging signatures. Replicative senescence is related to the finite cell proliferative capacity due to telomere shortening, while stress-induced premature senescence is independent of telomere shortening (41, 42). In this context, the enrichment in DNA damage pathways in CD4^+^ T cells from BC patients is consistent with the notion that this population is stimulated in the presence of tumor environmental cues that act as stress inducers.

Nowadays, data on the effector function of senescent T cells in cancer patients are scarce. Peripheral blood and tumor-infiltrating CD28^−^CD8^+^ T cells from cancer patients were shown to produce IL-10 (34). Also, CD28^−^Tim-3^+^CD4^+^ T cells from patients with hepatocellular carcinoma were reported to exhibit impaired ability to produce of IL-2 and IFN*γ* but their possible exhausted status was not ruled out (43). More recently, Egelston et al. (44) demonstrated that CD8^+^ TILs from breast cancer patients maintain cytokine production and degranulation ability and kill target cells regardless of their PD-1 expression. In contrast, PD-1^+^CD8^+^ TILs from patients with melanoma are functionally exhausted (45). Our characterization underscores that far from being dysfunctional, senescent T cells in BC patients retain robust capacity to produce effector cytokines. Interestingly, senescent CD4^+^ T cells exhibit high expression of granzymes, perforins, and CD107a, even comparable to CD8^+^ T cells. Moreover, senescent CD4^+^ T cells exhibit upregulation of numerous genes related to cytotoxicity (GZMH, GZMA, PRF1, EOMES, TBX21, CRTAM), supporting the hypothesis that they may represent a CD4^+^ CTL population. In this sense, CD4^+^ CTLs have been identified during aging, chronic viral infections, and in anti-tumor responses (46–48). Interestingly, a report by Phetsouphanh et al. (49) demonstrated that CD57-expressing CD4^+^ cytotoxic T cells might act together with CD8^+^ CTLs to control HIV viraemia in Elite controllers.

Our results agree with the observation that human CD4^+^ CTLs are enriched in the CD4^+^ T EMRA subset, most notably in donors with previous infection of dengue or cytomegalovirus (47). In fact, single cell transcriptome studies of the CD4^+^ T_EMRA_ lymphocytes identified cells with features of terminal CD4^+^-CTL effector phenotype (KLRG-1^high^, CD28^low^, CD27^low^). Moreover, Peguillet et al. (50) reported that CD25^−^CD127^−^CD4^+^ T cells are expanded in blood from patients with uveal melanoma and breast cancer patients undergoing neo-adjuvant chemotherapy compared to healthy donors. These CD4^+^ T cells are highly differentiated effector cells and display cytotoxic features. Interestingly, during neo-adjuvant chemotherapy in patients with breast cancer the increase of CD25^−^CD127^−^CD4^+^ T cells correlated with tumor regression.

More recently, Cachot et al. (51) confirmed the presence of cytolytic tumor-specific CD4^+^ T cells by mining single cell RNA-seq datasets from melanoma patients. When validating these data, the authors demonstrated that these tumor-specific CD4^+^ T cells are able to kill tumor cells by a direct contact and granzyme-dependent mechanism, but with a delayed kinetic when compared to a more classical CTL.

The contribution of senescent CD4^+^ T cells to the clinical outcome of patients remains unknown. In this work, we observed that higher expression of the signature genes CD4, KLRG-1, and B3GAT1 correlated with a higher survival rate when we analyzed the OS of patients from two invasive breast carcinoma cohorts from the TCGA consortium. We speculate that the higher survival rate could be attributed to the potential cytotoxic capacity of DP CD4+T. However, in the era of adoptive cell transfer, the presence of high frequencies of cell-cycle arrested T cell populations (CD4^+^ as well as CD8^+^) could be considered as a limiting factor for candidates for this type of immunotherapy. Considering that p38 activation by AMPK and TAB1 has been described as drivers of senescence in human T cells (52), it would be interesting to determine whether expansion of TILs or peripheral blood T cells to generate CAR-T cells in the presence of p38 inhibitors can recover the proliferative capacity of senescent T cells retaining their high effector function.

The analysis of a bigger cohort of BC patients could help identify if senescent T cells may be associated with disease outcome, prognosis, or response to treatment. Moreover, our data suggest that increasing the number of senescent T cells in peripheral blood of cancer patients should be taken into account when designing adoptive cell therapies using PB-derived T cells.

## Data Availability Statement

The datasets presented in this study can be found in online repositories. The names of the repository/repositories and accession number(s) can be found in the article/**Supplementary Material**.

## Ethics Statement 

This study was approved by the ethical Committee from Hospital Rawson-Provincia de Cordoba-Argentina, (approval#552014) and Hospital Curie- Paris- France (approval#DATA210006). All studies were conducted following institutional ethical guidelines and in accordance with the principles expressed in the Declaration of Helsinki. Written informed consent was obtained from all individuals to participate in this study.

## Author Contributions

MR designed the study, conducted experiments, analyzed and interpreted the data, and wrote the first draft of the manuscript. NN, CA, SB, FC, and CS participated in experiments. JT, WR, EP, MM participated in microarray experiments. CT and DC performed the analysis with public data. AC, ML, NP, and SV recruited breast cancer patients, collected samples at Rawson Hospital an Institute Curie and helped in the design of patient’s inclusion/exclusion criteria. EA, AG, and EP participated in data discussion, interpretation of results and manuscript revision. CM designed and supervised the study, analyzed and interpreted the data and wrote the manuscript. All authors contributed to the article and approved the submitted version.

## Funding

This work was supported by grants from PICT 2015-1954, SECYT 2012-2016, INC-Ministerio de Salud de la Nación Argentina (2015-2017) to CLM. FPC, MCR, and SNB were supported by fellowships from CONICET. NGN was supported by a fellowship from Ligue Nationale Contre le Cancer AG, EVAR and CLM are members of the Scientific Career in CONICET. NP is a member of the Technical Assistant Career from CONICET. The TransImm team is supported by the SiRIC-Curie Program (grant INCa-DGOS-12554), the LabEx DCBIOL (ANR-10-IDEX-0001-02 PSL, and ANR-11-LABX-0043), and the Center of Clinical Investigation (CIC IGR-Curie 1428).

## Conflict of Interest

The authors declare that the research was conducted in the absence of any commercial or financial relationships that could be construed as a potential conflict of interest.

## Publisher’s Note

All claims expressed in this article are solely those of the authors and do not necessarily represent those of their affiliated organizations, or those of the publisher, the editors and the reviewers. Any product that may be evaluated in this article, or claim that may be made by its manufacturer, is not guaranteed or endorsed by the publisher.

## Supplementary Material

Supplementary Table 1 Description of clinical and pathological characteristics of patients recruited and analyzed in the manuscript.

Supplementary Table 2 List of antibodies used for flow cytometry staining in the manuscript.

Supplementary Figure 1 Senescent CD4^+^ T cells do not proliferate. Representative zebra plots from sorted KLRG-1^−^CD57^−^, KLRG-1^+^CD57^−^ and KLRG-1^+^CD57^+^ CD4^+^ T cells from peripheral blood of breast cancer patient show the frequency of Ki-67^+^ cells after 72 h of anti-CD3/CD28 stimulation.

Supplementary Figure 2 KLRG-1^+^CD57^+^ CD8^+^ cells from peripheral blood exhibit features of senescence. **(A)** Pie charts exhibit proportion of CD27^+^CD28^+^ (white), CD27^+^CD28^−^ (light gray), CD27^−^CD28^+^ (dark gray) and CD27^−^CD28^−^ (black) within CD8^+^ T cell subpopulations defined by the expression of KLRG-1 and CD57, for all HDs and BC patients analyzed. **(B)** Bar graph shows frequency of CD27^−^CD28^−^ cells within KLRG-1^+^CD57^+^ CD8^+^ T cells from HDs (dots) and BC patients (triangles). **(C)** Mean frequency (± SEM) of SA-*β*gal^+^ CD8^+^ T cells within DN, SP, and DP subsets. **(D)** Mean frequency (± SEM) of **γ**H2AX^+^ cells in DN, SP, and DP CD8^+^ T cell subsets. **(E)** Bar graph shows frequency of **γ**H2AX^+^ cells within KLRG-1^+^CD57^+^ CD8^+^ T cells from HD (dots) and BC patients (triangles). **(F)** Bar graph shows frequency of proliferating (Ki-67^+^) CD8^+^ T cells after stimulation and within CD57^−^ and CD57^+^ populations from BC patients. **(G)** Mean frequency (± SEM) of IL-2^+^ cells, in DN, SP, and DP CD8^+^ T cells. In all cases, each dot/triangle/square represents a subject analyzed. For two group comparisons (HD *vs* BC) unpaired *t*-tests were used (*p* is indicated in each graph). For three groups comparisons (DN, SP, and DP) matched one-way ANOVA and Tukey multiple comparisons tests were used (**p* < 0.05; ***p* < 0.01; ***p < 0.005; ****p < 0.001; *ns*, not significant).

Supplementary Figure 3 Frequency of T cell subpopulations within CD4^+^ and CD8^+^ T cells. Graphs show frequency of naive, CM, EM, and EMRA cells within total CD4^+^ or CD8^+^ T cells from HDs (dots) and BC patients (triangles). Each dot/triangle represents a subject analyzed. One-way ANOVA and Tukey multiple comparisons tests were used to compare subsets from HD *vs.* BC (*ns*, not significant; **p* < 0.05).

Supplementary Figure 4 Senescent CD8^+^ T cells from peripheral blood exhibit an effector-memory/EMRA phenotype express high levels of inhibitory receptors and are highly polyfunctional. **(A)** Bar graphs show proportion of naive (white), CM (light gray), EM (dark gray) and EMRA (black) cells within DN and DP CD8^+^ T cell subpopulations, for all BC patients and HDs analyzed. **(B)** Line graphs show frequency of iR-expressing CD8^+^ T cells within EM/EMRA populations and within DN and DP subsets as indicated in graphs. Each line represents a subject analyzed. **(C)** Bar graphs show iR-expressing CD8^+^ T cells within KLRG-1^+^CD57^+^ EM/EMRA population from BC patients and HDs. Paired *t*-tests were used to compare iR expression between DN and DP subsets **(B)** or between HD and BC patients **(C)** (**p* < 0.05; ***p* < 0.01; ****p* < 0.005; absence of *p-*values indicates no statistical differences). **(D)** Line graphs show frequency of BC patients’ CD8^+^ T cells co-expressing TNF and IFN**γ** or Granzyme B and CD107a (after PMA/Ionomycin stimulation) or expressing Perforin (*ex vivo*), within EM/EMRA populations and within DN and DP subsets as indicated in graphs. Each line represents a subject analyzed. Paired *t*-tests were used (*p* are indicated in each graph). **(E)** Bar graphs show proportion of KLRG-1^+^CD57^+^CD8^+^ T cells expressing the indicated cytokines or cytotoxic-associated molecules in HD and BC patients. Unpaired *t*-tests were used to compare HD *vs.* BC (ns, not significant).

Supplementary Figure 5 Senescent CD4^+^ T cells from invaded/metastatic tumor-draining lymph nodes express inhibitory receptors and are highly polyfunctional cells. Line graphs show frequency of CD4^+^ T cells from invaded/metastatic tumor-draining lymph nodes, expressing PD-1 and TIGIT **(A)** or co-expressing TNF and IFN**γ** or expressing CD107a **(B)** (after PMA/Ionomycin stimulation) within EM/EMRA populations and within DN and DP subsets as indicated in graphs. Each line represents a sample analyzed. Paired *t*-tests were used to compared DN and DP subsets (*p* are indicated in each graph).

Supplementary Figure 6 Gene expression profiles of CD4^+^ T subsets from peripheral blood from BC patients and HDs. **(A)** Principal component analysis of all analyzed groups. Each dot represents a different sample, and each color represents a group as indicated. **(B)** Heat maps show the relative gene expression of cytokine/chemokine network and T-cell signaling network of DPBC and DNBC samples. **(C)** GSEA plot shows the enrichment of the gene set GSE: 13738 in the transcriptome of DPBC *vs* DNBC. **(D)** Graph shows the p-value of selected pathways significantly enriched in DEGs of panel **(A)** (DPBC *vs* DPHD) using EnrichR. **(E, F)** GSEA plots show the enrichment of the gene set E GSE: 26495, F GSE: 11057 in the transcriptome of DPBC *vs* DPHD. In all enrichment plots, normalized enrichment score (NES) and significant *p-*value (FCRq) are indicated.

## Abbreviations

BC, breast cancer; DEG, differentially expressed genes; DN, double negative cells KLRG-1^−^CD57; DP, double positive cells KLRG-1^+^CD57; EMRA, terminally differentiated effector memory; GSEA, gene set enrichment analysis, HD, healthy donors; *γ*H2AX, phosphorylated form of histone H2AX; KLRG-1, killer cell lectin-like receptor subfamily G member 1; LN(s), lymph node(s); NI-LN(s), non-invaded lymph node(s); PBMCs, peripheral blood mononuclear cells; PCA, principal component analysis; PMA, phorbol myristate acetate; SA-*β*gal, senescence-associated *β* galactosidase; SASP, senescence-associated secreting phenotype; SP, simple positive cells KLRG-1^+^CD57^−^; TIL, tumor-infiltrating lymphocytes; Tregs, regulatory T cells Foxp3^+^.
