# Supplementary material for: Polyfunctional KLRG-1+CD57+ Senescent CD4+ T Cells Infiltrate Tumors and Are Expanded in Peripheral Blood From Breast Cancer Patients
Source: Front Immunol. 2021 Jul 27;12:713132. doi: 10.3389/fimmu.2021.713132 (PMC8353459; doi:10.3389/fimmu.2021.713132)
Supplement: Supplementary Table 1 — Description of clinical and pathological characteristics of patients recruited and analyzed in the manuscript. [file Table_1.pdf]

Table S1. Clinical and Pathological data of recruited/analyzed Breast Cancer Patients	
**Characteristics**	Patients (#)
**Recruited/analyzed**	43
**Histology**	
Invasive ductal carcinoma (IDC)	33
Invasive lobular carcinoma (ILC)	5
Others (mixed, papillary, medullary, mucinous)	5
**Stage**	
I	12
II	20
III	8
IV	0
**Histological grade (Easton and Elis)**	
Well differentiated (I)	0
Moderately differentiated (II)	14
Poorly differentiated (III)	25
**Tumor size (cm)**	
T1 (≤2)	13
T2 (2-5)	26
T3 (>5)	4
**Hormone receptor status**	
Estrogen receptor (ER)	34
Progesterone receptor (PR)	29
HER2	8
**Lymph node status**	
Free (N0)	18
N1 (1-3)	13
N2 (4-9)	6
N3 (>9)	2

Stage for 3 patients was not available
Histological grade for 4 patients was not available
Lymph node status for 4 patients was not available
