# Supplementary material for: Polyfunctional KLRG-1+CD57+ Senescent CD4+ T Cells Infiltrate Tumors and Are Expanded in Peripheral Blood From Breast Cancer Patients
Source: Front Immunol. 2021 Jul 27;12:713132. doi: 10.3389/fimmu.2021.713132 (PMC8353459; doi:10.3389/fimmu.2021.713132)
Supplement: Supplementary Table 2 — List of antibodies used for flow cytometry staining in the manuscript. [file Table_2.pdf]

Table S2. Antibodies used.

Antigen	Clone	Fluorochrome	Company
**Live/Dead**	Fixable	Aqua	Life Technologies
	No fixable	DAPI	BD
**CD45**	2D1	APC-Cy7	BD
**CD3**	SK7	PerCP	BD
	OKT3	PerCP // Brillant Violet 650	Biolegend
	UCHT1	FITC	ImmunoTech
	UCHT1	Alexa Fluor 700	BD
	UCHT1	APC-Cy7	Biolegend
**CD8**	RPA-T8	PerCP-Cy5.5 // APC	eBioscience
	RPA-T8	APC-R700	BD
	RPA-T8	Alexa Fluor 700	Biolegend
	3B5	Alexa Fluor 700	Life Technologies
	HIT8a	PE-Cy7	Biolegend
**CD4**	M-T466	FITC	Miltenyi Biotec
	RPA-T4	PE // PerCP-Cy5.5 // PE-Cy7	eBioscience
	RPA-T4	Alexa Fluor 700	Biolegend
	OKT4	Brillant Violet 785	Biolegend
	HI30	APC	BD Pharmigen
**CD197 (CCR7)**	150503	Biotin	BD
	3D12	PE // PE-Cy7	eBioscience
	3D12	BV421	BD
	G043H7	PerCP-Cy5.5	Biolegend
**KLRG1**	13F12F2*	PE-Cy7 // APC // PerCP-eFluor 710	ThermoFisher
	REA261	APC-Vio770	Miltenyi Biotec
**CD57**	HCD57	PE-CF594	BD Pharmigen
	HCD57	PE	Biolegend
**CD45RA**	HI100	Brillant Violet 650	Biolegend
	HI100	PE-Cy5 // PE-Cy7	BD Pharmigen
	ALB11	PE	Immunotech
**CD27**	O323	Brillant Violet 605 // Alexa Fluor 700	Biolegend
	O323	FITC // APC-eFluor 780	eBioscience
	O323	PE	BD
	L3D10	APC	BD
**CD28**	CD28.2	PerCP-Cy5.5 // FITC // PE-Cy7	Biolegend
	CD28.2	APC	eBioscience
	CD28.2	PE	BD
**CD25**	M-A251	PE	BD Pharmigen
**CD244 (2B4)**	C1.7	PE // APC	Biolegend
**CD160**	BY55	PerCP-Cy5.5	Biolegend
**CD272 (BTLA)**	J168-540	PE-CF594	BD
	MIH26	APC	Biolegend
**CD279 (PD-1)**	EH12.2H7	APC // Brillant Violet 421	Biolegend
	EH12.1	PE	BD
	J105	PE // PE-Cy7	eBioscience

**CD155 (TIGIT)**	A15153G	PE/Dazzle™ 594	Biolegend
	MBSA43	FITC // PerCP-eFluor 710	eBioscience
**CD107a**	eBioH4A3	FITC	eBioscience
**FoxP3**	236A/E7	PE // Alexa Fluor 488 // PE-Cy7	eBioscience
**γH2AX**	N1-431	Alexa Fluor 647	BD
**TNF**	MAB11	APC // PE	eBioscience
**IFN-γ**	B27	V450	BD
	B27	FITC	BD
	B27	FITC	Biolegend
**IL-2**	MQ1-17H12	PE // APC	BD
**Granzyme B**	GB11	FITC	BD
	GB11	PE	eBioscience
	GB11	Alexa Fluor 647	Biolegend
**Perforin**	B-D48	PE	Biolegend
**BrdU**	BU20A	PE	BD
**Ki-67**	SOL A15	APC	eBioscience
**EpCAM**	1B7	PerCP-eFluor 710	eBioscience

*Some experiments were performed with clone 13F12F2 kindly provided by Dr. Pircher (Germany) and some other with the later 13F12F2 clone commercially available as indicated.
